# Age, size and body condition do not equally reflect population response to habitat change in the common spadefoot toad *Pelobates fuscus*

**DOI:** 10.7717/peerj.11678

**Published:** 2021-07-15

**Authors:** Dan Cogălniceanu, Florina Stănescu, Diana Székely, Theodor-Sebastian Topliceanu, Ruben Iosif, Paul Székely

**Affiliations:** 1 Faculty of Natural and Agricultural Sciences, Ovidius University Constanța, Constanța, Romania; 2 Asociația Chelonia Romania, Bucharest, Romania; 3 Black Sea Institute for Development and Security Studies, Ovidius University Constanța, Constanța, Romania; 4 CEDMOG-Center for Morphological and Genetic Studies of Malignant Pathology, Ovidius University Constanța, Constanța, Romania; 5 Departamento de Ciencias Biológicas y Agropecuarias, Laboratorio de Ecología Tropical y Servicios Ecosistémicos (EcoSs Lab), Universidad Técnica Particular de Loja, Loja, Ecuador

**Keywords:** Amphibia, Fitness, Growth, Habitat fragmentation, Habitat loss, Life history, Monitoring, Romania, Skeletochronology, Urbanization

## Abstract

Urbanization impacts biodiversity both directly through physical expansion over land, and indirectly due to land use conversion and human behaviors associated with urban areas. We assessed the response of a common spadefoot toad population (*Pelobates fuscus*) to habitat loss and fragmentation resulting from urban development by studying changes in size, body condition and age parameters. We compared samples collected in the early 2000s (sample A) and later on during 2012–2014 (sample B). The terrestrial habitats in the study area were severely reduced and fragmented due to the expansion of the human settlement. We found no significant differences in the age parameters between the two sampling periods; the median lifespan shortened from 3.5 (sample A) to 3.0 years (sample B), while the other age parameters were similar in both samples. In contrast, snout-vent length, body mass and body condition experienced a significant decrease over time. Our results suggest that changes in body size and body condition, rather than age parameters, better reflect the response of the common spadefoot toad population to declining habitat quality. Therefore, body measurements can provide reliable estimates of the impact of habitat degradation in amphibian populations.

## Introduction

Amphibians are suffering a severe worldwide decline, being the most affected group of terrestrial vertebrates, with nearly one-third (32.5%) of the species threatened (Houlahan et al., 2000; Stuart et al., 2004; McCallum, 2007; Collins & Crump, 2009; Baillie et al., 2010). The main responsible factors are habitat fragmentation and destruction, climate change, UV-B radiation level increase, chemical pollution, pathogens, alien species, and over-exploitation (e.g., Young et al., 2001; Baillie et al., 2010; Vitt & Caldwell, 2014; Nunes et al., 2019; Falaschi et al., 2020). These stressors induce a series of direct or indirect changes upon amphibians’ phenology, behaviour, physiology, metabolism, and depending on their severity, may cause population declines and local or regional extinction (e.g., Sodhi et al., 2008; Hoffmann & Sgrò, 2011; Blaustein et al., 2012; Pimm et al., 2014; Catenazzi, 2015; Grant et al., 2016).

Urbanization is a recently recognized key driver of global environmental change that impacts biodiversity, both directly through physical expansion over land, and indirectly due to land use conversion and human behaviors associated with urban areas (Chace & Walsh, 2006; Elmqvist et al., 2013; Brice, Pellerin & Poulin, 2017). Habitat loss and degradation caused by spatial expansion can eliminate organisms outright, but mainly they alter the conditions that a species requires to survive. There is increasing evidence that, along with direct effects in the form of habitat loss and fragmentation, negative impacts of urbanization on biodiversity extend to pollution (chemical, light, noise), microclimate change, introduction of alien species and intensification of traffic (Elmqvist, Zipperer & Güneralp, 2016). Urbanization was also found to cause a decline in species richness in several taxonomic groups (McKinney, 2008; aquatic macroinvertebrates: Thornhill et al., 2017; amphibians and reptiles: Cordier et al., 2021), as well as changes in ecological processes (Miles et al., 2019, Fisogni et al., 2020).

A variety of studies have confirmed that landscape changes have a negative impact on amphibian populations, affecting their abundance (e.g., Pellet, Guisan & Perrin, 2004; Pillsbury & Miller, 2008), age structure (Bionda et al., 2018; Jennette, Snodgrass & Forester, 2019), and body condition (Janin, Léna & Joly, 2011). Due to reduced habitat quality, amphibian populations under urban pressure show in some cases smaller individual body size and poorer body condition compared to more natural ecosystems (e.g., Jennette, Snodgrass & Forester, 2019; Bókony et al. 2018; Lunghi et al., 2018; Kärvemo, Laurila & Höglund, 2019), although the magnitude and even the direction of the response varies according to species resilience (Iglesias-Carrasco, Martín & Cabido, 2017; Murphy et al., 2016). As organisms with indeterminate growth, amphibians adopt a diversity of life-history strategies, which usually entail trade-offs in resource allocation between growth and reproduction, in order to maximize reproductive success and survival in response to a given environmental variation (e.g., Heino & Kaitala, 1999; Fox, Roff & Fairbairn, 2001; Cogălniceanu & Miaud, 2003; Morrison & Hero, 2003; Iturra-Cid, Ortiz & Ibargüengoytía, 2010; Hjernquist et al., 2012). Temperate amphibians have a complex bi-phasic life-cycle, and the most evident trade-off between the aquatic and terrestrial stages is reflected in body size at metamorphosis, effects of aquatic stressors being carried over into terrestrial growth, survival, and performance (Székely et al., 2020; Thompson & Popescu, 2021).

These trade-offs are ultimately reflected in the individual fitness, and affect growth and demographic parameters, like age structure (Roff, Heibo & Vøllestad, 2006). Therefore, these parameters can be used to detect population responses to a changing environment (e.g., Karraker & Gibbs, 2011; Zhang & Lu, 2012; Amat & Meiri, 2018). A deteriorated environment associated to anthropogenic disturbance can determine reductions in body size either because individuals lack the adequate resources necessary for growth, or are confronted with disease and contaminants (Kärvemo, Laurila & Höglund, 2019), or because a larger proportion of the population consists of younger individuals due to decreased longevity (Bionda et al., 2018).

Body condition is considered both a measure of individual fitness and an indicator of environmental stress (e.g., Băncilă et al., 2010), since it is related to a series of internal and external factors such as metabolism (MacCracken & Stebbings, 2012), health (Ullman-Culleré & Foltz, 1999), energy reserves (Denoël et al., 2002; Schulte-Hostedde et al., 2005), food availability and habitat quality (Sztatecsny & Schabetsberger, 2005; Janin, Léna & Joly, 2011; Scheele et al., 2014; Unglaub et al., 2018), and climatic factors (Reading, 2007). Thus, changes in body condition can provide valuable information regarding the efficiency of the life strategy adopted by a population in a given environment.

Skeletochronology, alongside capture-mark-recapture studies, is a widely used non-lethal method to estimate age-related parameters in amphibians (Smirina, 1994; Sinsch, 2015). Individual age can be assessed based on the presence of annual lines of arrested growth (LAGs) deposited in the bone tissue during periods of inactivity like hibernation and aestivation (Smirina, 1994; Sinsch et al., 2007). Additionally, changes in the rate of growth as a result of transitions from a life-stage to another (for example reaching sexual maturity) also affect the bone structure, allowing an estimation of the age when an individual becomes adult (Smirina, 1994). Age and size at which transitions occur often change plastically in response to environmental conditions (Roff, 1992), and their study allows us to determine population responses and trends over time. As such, age-related parameters obtained by skeletochronology proved valuable tools when studying population responses to environmental stress like habitat loss and degradation (Middleton & Green, 2015), urbanization (Yetman, Mokonoto & Ferguson, 2012), pollution (Spear et al., 2009; Kaczmarski et al., 2016; Otero et al., 2018), parasitic infestation (Gustafson et al., 2015; Sinsch, Kaschek & Wiebe, 2018), or pathogens (Campbell et al., 2018). They were also successfully used to evaluate performance and well-being of amphibian populations inhabiting differently-managed landscapes (e.g., Orchard, Tessa & Jehle, 2019), or to understand the effects of habitat quality on amphibian life histories (Sinsch et al., 2007).

We assessed the response of a common spadefoot toad population to habitat loss and fragmentation caused by urban development (i.e., increase of associated transport infrastructure and constructed areas) over more than a decade. We assessed changes in age, size, and body condition as proxies of resilience to habitat loss and fragmentation. We hypothesized that the population would respond through (1) decrease in size and body condition, and (2) decrease in average lifespan and longevity. Although the sampling was not continuous during the study timeframe, we expected to find significant changes of life-history parameters, given the relatively short average life-span of the studied species of 5 years (Cogălniceanu et al., 2014).

## Materials & methods

### Study species

The common spadefoot toad *Pelobates fuscus* (Laurenti, 1768) is a widespread species ranging throughout most of Europe (Dufresnes et al., 2019a; 2019b). It is a highly specialized, nocturnal, burrowing anuran, with strong population declines due to habitat loss (Eggert et al., 2006; Temple & Cox, 2009). Breeding occurs in permanent or temporary ponds. The breeding season is short, lasting up to 1–2 weeks in the early spring, followed by a long larval stage (Cogălniceanu et al., 2014). Landscape features have a strong influence on the spadefoot toads’ distribution because they have low dispersal abilities, and adults spend most of their terrestrial life close to the breeding ponds (Nielsen & Dige, 1995). During their terrestrial life, suitable habitat for the species is represented by open landscapes with loose, sandy soils, including pasturelands and cultivated fields, forested areas with >75% canopy cover and scrubby areas being avoided, and roads with medium and high traffic loads representing important dispersal barriers in addition to causing high mortality (Eggert, 2002, Nyström et al., 2002).

### Study area & habitat changes over time

The study was conducted in northwestern Romania, at the outskirts of Sălicea, Cluj County, Romania (46°40′58.4″ N; 23°32′38.8″ E, 720 m a.s.l.). Since 2008, the locality is part of one of the 19 territorial units of the metropolitan area of Cluj–Napoca, the fourth most populous city in Romania. The population decreased during 1995–2008 and increased during 2009–2016, but the built-up areas for both primary and secondary residence, as well as entertainment and lodging infrastructure grew constantly since 1995.

**Figure 1:**
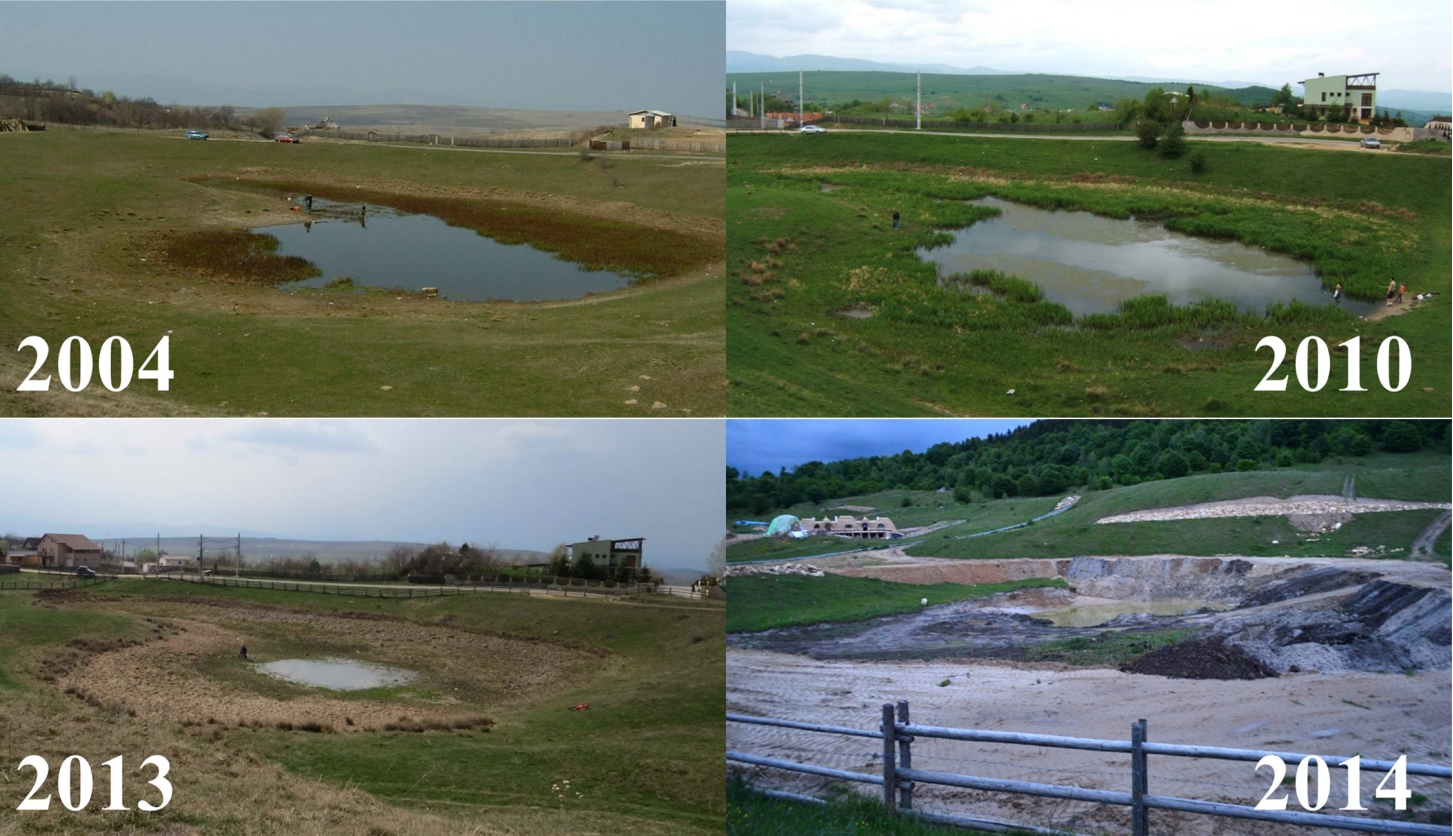
Changes of the breeding pond and surrounding area over a decade. Photo credit: Diana Székely.

The study site consisted of a freshwater permanent pond originally surrounded by steppe grasslands and forests, situated in the vicinity of a medium traffic interprovincial/county road (Fig. 1). This was the only suitable breeding site in the whole area where large numbers of spadefoot toads were observed and thus we can assume that no immigration occurred from nearby areas. Most specimens were caught in and around the pond of approximately 2,700 m^2^, whose area varied over the years depending on the amount of rainfall and snow. Besides *P. fuscus*, the pond was also used as a breeding site by *Lissotriton vulgaris* (Linnaeus, 1758), *Triturus cristatus* (Laurenti, 1768), *Bufo bufo* (Linnaeus, 1758), *Hyla arborea* (Linnaeus, 1758), *Rana temporaria* (Linnaeus, 1758), and *Rana dalmatina* (Fitzinger, 1838).

We evaluated habitat changes within a radius of 300 m from the centre of the breeding pond, in order to quantify the reduction in the availability and quality of surrounding terrestrial habitats. We chose this distance according to the average seasonal/daily movement recorded for this species (Glandt, 1986; Eggert, 2002) and because this species was shown to exhibit a high degree of breeding site fidelity (Hels, 2002). We extracted satellite images available in Google Earth Pro V. 7.3.3.7786 at three different moments in time, relevant for our study timeframe: the first image was taken on March 2003 (Google Earth, 2003), the second on May 2011 (Google Earth, 2011) and the third on August 2014 (Google Earth, 2014). We re-georeferenced the three images in a GIS environment and digitized the landscape features at an eye altitude of 1.8 km. We extracted the area covered by four landscape features, namely forest, pasture, transport and urban. Forest includes compact patches of deciduous trees dominated by *Quercus* spp. but also fragments of shrubs dominated by *Corylus avellana* and *Rubus* spp. We digitized pasture features as open habitat patches known for seasonal grazing. Transport includes all transport infrastructure (i.e., both paved and unpaved, secondary roads), while urban includes a mosaic of constructed areas (i.e., buildings, yards, construction sites, associated paved surfaces other than roads). The area occupied by each type of landscape feature was expressed as a percentage.

### Sampling

Sampling was performed each year in April, during the short breeding season, at two moments in time: between 2000 and 2004 (hereafter moment A, sample A), and between 2012 and 2014 (hereafter moment B, sample B). Sampling and measurements were done by the same two researchers (DS and PS), using the same methods, during both study moments (A and B). They performed Visual Encounter Surveys in and around the pond between 19:00–02:00 and caught the frogs by hand or with a dip net. Only adult individuals were included in our study. Sex was determined based on the presence/absence of the humeral glands which are typical to sexually mature males. Snout-vent length (SVL) was measured with dial calipers at 0.1 mm precision and body mass (BM) with a portable electronic balance at 0.1 g precision, for a total of 279 individuals (120 females, 159 males) in sample A, and 94 individuals (42 females, 52 males) in sample B (Table S1). Females were weighed before egg deposition. The longest toe of the right forearm was clipped and stored in 70% alcohol from 58 individuals (no sex and SVL data assigned) in sample A, and 94 individuals (42 females and 52 males) in sample B. Sex and size data was assigned to each collected toe only in sample B, but not A. At moment A, toe-clipping was only used for the purpose of individual identification and only a small part of these toes was preserved. Therefore, we could not account for sex when we estimated and compared age parameters between the two sampling moments, nor compare growth based on size-at-age data between the two periods.

The methods used in this study received full approval from Ovidius University Constanţa through the Ethics Committee of the Faculty of Natural and Agricultural Sciences. Field sampling was approved through Ministerial Order no. 1173/2010.

### Skeletochronology and age-related parameters

We estimated age from the collected toes using skeletochronology, based on the protocol of Castanet & Smirina (1990) with slight modifications, as described in Stănescu et al. (2016). The bone tissue was decalcified in 5% nitric acid, rinsed and kept in distilled water overnight, followed by inclusion in Tissue-Tek® O.C.T.™ Compound (Sakura Finetek, Torrance, CA, USA). We cut fine cross-sections (12–14 μm) using a freezing microtome (Tehsys, CA, USA 3,000 CR) and stained them in Ehlrich’s haematoxylin. Cross-sections with the narrowest marrow cavity and the widest cortical bone were permanently mounted on slides using Aquatex® (aqueous mounting agent for microscopy, Merck Millipore, Burlington, MA, USA) and photographed using an Olympus® E-620 microscope-mounted camera (CX 31 microscope, Olympus®, Shinjuku City, Tokyo, Japan) and Quick Photo Micro 2.3 software. Three independent observers (FS, DC and ST) counted the LAGs in 3–5 sections per individual. Each LAG was considered to represent 1 year of age.

We computed the following age-related parameters for each of the two samples (A and B): 1. average lifespan (mean and median age); 2. longevity (i.e., maximum age observed); 3. age at sexual maturity, considered as the youngest age class in our sample, either observed or inferred from the bone growth pattern following Smirina (1994); 4. potential reproductive lifespan (i.e., time span between the age at sexual maturity and the maximum age observed in the sample). In addition, we computed: 5. the annual adult survival rate (*S*) according to Robson and Chapman’s formula (see Miaud, Guyétant & Elmberg, 1999): }{}S = T/\left( {R + T - 1} \right), where }{}T = {N_1} + 2{N_2} + 3{N_3} + 4{N_4} + \ldots ,\; R = \sum {N_i}, *N*_*i*_ = number of individuals in age group *i*; 6. adult life expectancy (*ESP*), the expected total longevity of individuals which have reached maturity, estimated using Seber’s formula (Seber, 1973): }{}ESP = 0.5 + 1/\left( {1 - S} \right). *ESP* is the expected average age and differs from the “longevity” value that is simply the highest recorded age, which can be affected by sample size since the probability of encountering older individuals increases with sample size.

Since sex and SVL data were only assigned to the bone samples collected at moment B, comparisons between males and females regarding age distribution and growth were only performed for this sample. We applied a von Bertalanffy’s growth model (von Bertalanffy, 1938) following Beverton & Holt (1957): }{}SV{L_t} = SV{L_{max}} \times \; \left( {1 - {e^{ - k\; \times \; \left( {t\; - \; {t_0}} \right)}}} \right), where SVL_t_ is the expected or average SVL at time (or age) *t*, SVL_max_ is the asymptotic average SVL, *k* is the growth rate coefficient and *t*_0_ is the time or age when the average SVL was zero. To calibrate the growth model, we used measurements of SVL at metamorphosis (i.e., 21.26 mm) provided by Stănescu et al. (2013). We fitted the von Bertalanffy’s growth model and estimated growth parameters (VBGPs) by nonlinear least squares, and two estimated VBGPs were considered significantly different at the 0.95 level when their confidence intervals (CI 95%) did not overlap (Stănescu et al., 2016). The growth model and parameters were estimated in R Studio v. 1.1.423 (R Core Team, 2017), with the packages FSA (Ogle, 2016) and nlstools (Baty et al., 2015).

### Size-related parameters

We computed the residual body condition index (BCI) based on the linear regression between log-transformed (log10) values of SVL and BM (e.g., Denoël et al., 2002; Băncilă et al., 2010). Since *P. fuscus* shows a significant sexual size dimorphism females being both larger and heavier than males, we computed the BCI separately for males and females. The BCI values had a normal distribution and fulfilled the required assumptions for a true measure of body condition (Blackwell, 2002; Schulte-Hostedde et al., 2005; Băncilă et al., 2010). We considered that positive BCI values indicated a good condition, while negative values indicated a poor condition of the sampled individuals (Jakob, Marshall & Uetz, 1996; Schulte-Hostedde et al., 2005; Blackwell, 2002).

We computed a sexual dimorphism index (SDI) of SVL and BM following Lovich & Gibbons (1992), with the results arbitrarily defined as positive when females are the larger sex and negative in the converse situation.

We performed the statistical analyses using IBM SPSS Statistics for Windows version 20.0 (IBM Corp., Endicott, NY, USA 2011). We tested all data for normality and homoscedasticity using Shapiro–Wilk and Levene tests respectively, and chose the subsequent statistical tests accordingly. The significance level was set at *α* = 0.05.

## Results

### Habitat change

**Figure 2:**
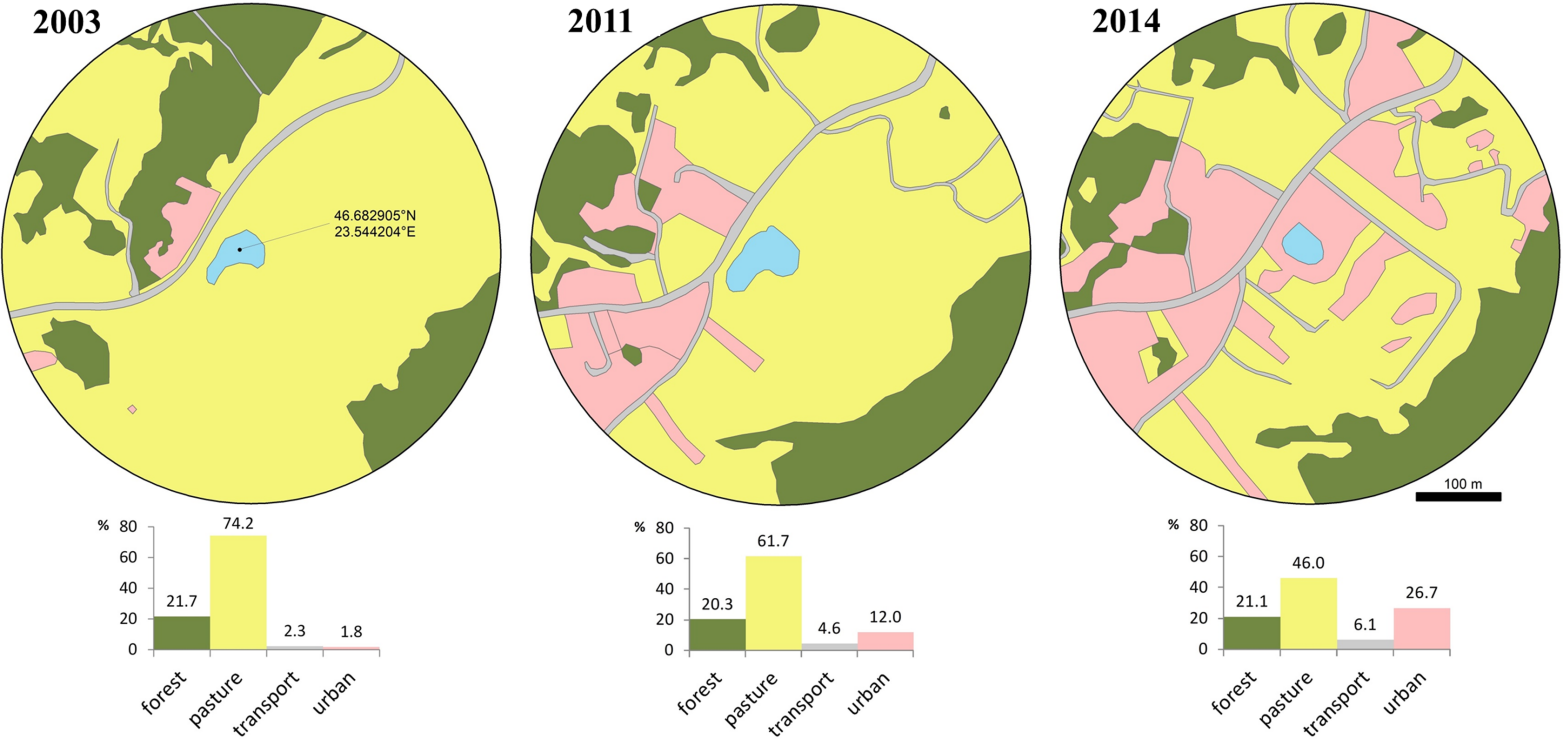
Loss of available terrestrial habitat between 2003–2014, caused by urban development within a 300 m radius from the breeding pond. Habitat cover is expressed as percentage at three moments (2003, 2011, 2014): forest, pasture, transport and urban. Transport includes all transport infrastructure (i.e., both paved and unpaved, secondary roads), urban includes all constructed areas (i.e., buildings, yards, construction sites, associated paved surfaces other than roads).

The favorable habitat (i.e., pasture) decreased from 74.2% to 46% between 2003 and 2014, while transport and urban areas increased from 4.1% to 32.8% (Fig. 2). During a short period of time (2011–2014) urban areas more than doubled. Access to the breeding site was more restricted and involved crossing secondary roads, with a higher mortality risk due to increased traffic in the area.

### Morphological parameters

**Figure 3:**
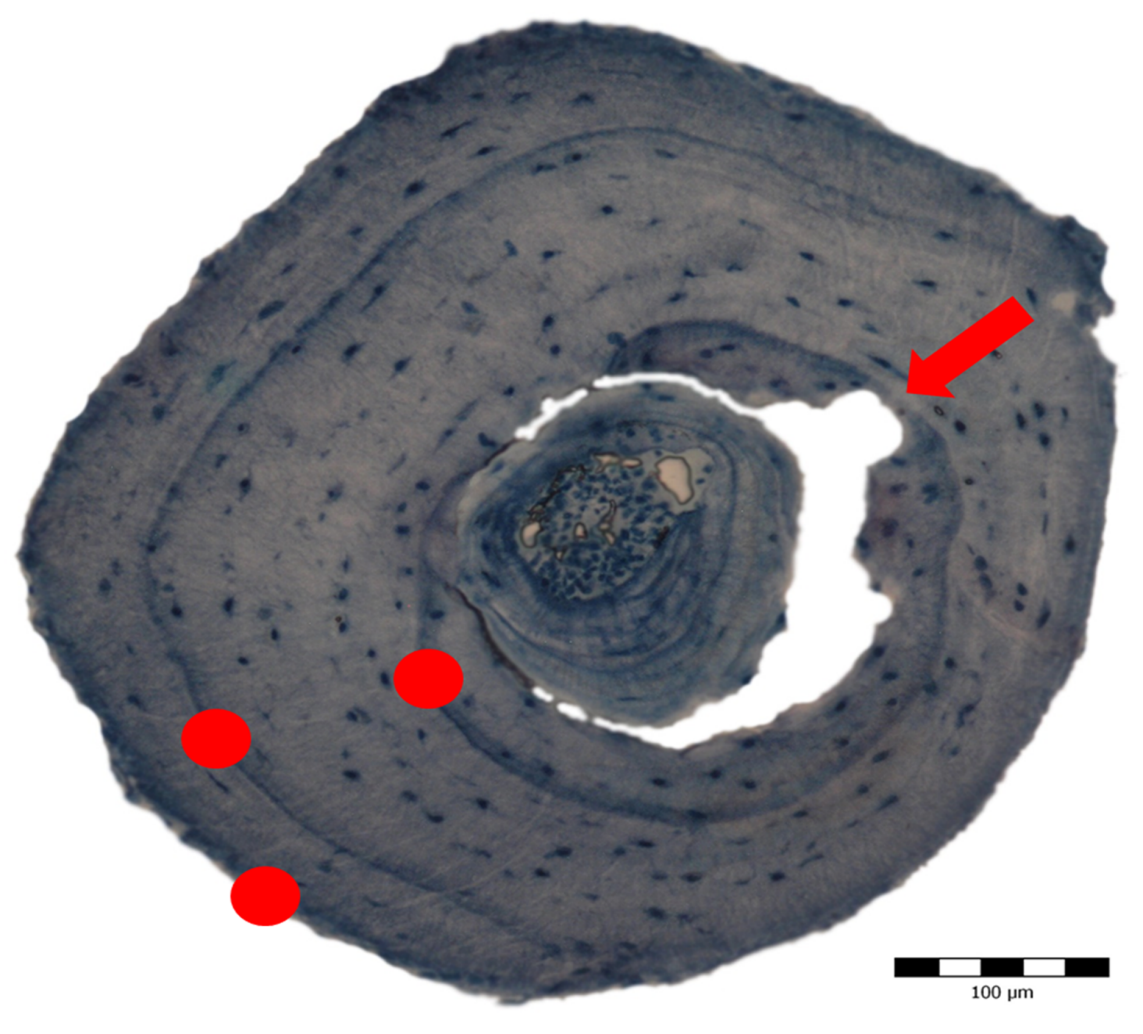
Cross section obtained through skeletochronology from the mid-diaphysis of a phalanx from a 3-year old adult *Pelobates fuscus* sampled in 2004. The red dots mark the lines of arrested growth (LAGs) deposited during hibernation, each corresponding to 1 year of life. The red arrow indicates the beginning of endosteal resorption.

Body size parameters are presented in Table 1. SVL and BM in both sexes were lower in sample B, compared to sample A (females: SVL, *t*_(53.5)_ = 2.385, *p* = 0.021, Cohen’s *D* = 0.51; BM, *t*_(52.9)_ = 2.647, *p* = 0.011, Cohen’s *D* = 0.57; males: SVL, *t*_(209)_ = 2.521, *p* = 0.012, Cohen’s *D* = 0.39; BM, *t*_(72.6)_ = 2.918, *p* = 0.005, Cohen’s *D* = 0.51) (Fig. 3). Individuals from sample B had a significantly lower body condition both in males (*t*_(208)_ = 2.826, *p* = 0.005, Cohen’s *D* = 0.45), and females (*t*_(160)_ = 2.673, *p* = 0.008, Cohen’s *D* = 0.47). Despite the decrease in body size and body condition, the SDI remained unchanged with females being larger (SDI_SVL_ was 1.128 in sample A and 1.124 in sample B) and heavier (SDI_BM_ was 1.492 in sample A and 1.476 in sample B) than males.

**Table 1:** Size-related parameters in the studied *Pelobates fuscus* population, at two sampling moments (A and B).

Sample	Sex	*n*	SVL (mm)	BM (g)
A (2000–2004)	Males	159	55.2 ± 3.5 45.0–63.0	21.3 ± 3.9 13.0–30.0
A (2000–2004)	Females	120	62.3 ± 3.9 52.0–72.0	31.8 ± 6.0 16.5–45.0
B (2012–2014)	Males	52 (51 in BM)	53.6 ± 4.3 42.8–59.9	19.1 ± 4.8 10.0–28.3
B (2012–2014)	Females	42	59.8 ± 6.2 45.9–75.6	27.6 ± 9.6 12.2–54.4

**Note:**

SVL = snout-vent length (mm); BM = body mass (g); *n* = sample size; values are provided as mean ± standard deviation and range (min-max). The sampling interval (years) is provided within parentheses for each sampling moments.

### Age-related parameters

**Figure 4:**
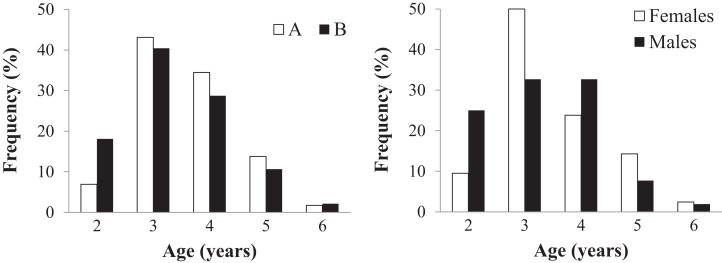
Age distribution of the studied *Pelobates fuscus* population, following skeletochronological assessment. Left - Age distribution for the pooled sample of males and females, at each of two moments in time–2000–2004 (sample A, white bars) and 2012–2014 (sample B, black bars). Right - Age distribution of males (black) and females (white) only within sample B (2012–2014).

Age could be assessed in all 152 individuals (58 in sample A and 94 in sample B) (Table 2 and Table S2). Endosteal resorption was observed in all samples, but did not interfere with LAG count (Fig. 4). There were no significant differences in the age distribution between the two sampling moments (Kolmogorov–Smirnov test: *Z* = 0.670, *p* = 0.760) (Fig. 4 left), nor between males and females from sample B (*Z* = 0.756, *p* = 0.634) (Fig. 4 right). The average lifespan was lower in sample B, but the difference was not significant (Mann–Whitney *U* = 2359, *Z* = −1.471, *p* = 0.141); the other age-related parameters were similar in both samples (Table 2).

**Table 2:** Age-related parameters in the studied *Pelobates fuscus* population, at two sampling moments (A and B).

Age-related parameters	Sample A	Sample B	Sample B	Sample B
	Males and females	Males and females	Males	Females
Sample size *n*	58	94	52	42
Median life span	3.5	3.0	3.0	3.0
Average life span ± SD	3.60 ± 0.88	3.38 ± 0.94	3.28 ± 0.99	3.5 ± 0.94
Age at sexual maturity	2 (85%) 3 (15%)	2 (93.3%) 3 (6.7%)	2 (97.6%) 3 (2.4%)	2 (88%) 3 (12%)
Longevity	6	6	6	6
Potential reproductive lifespan	4	4	4	4
Annual survival rate (S)	0.62	0.58	0.56	0.60
Adult life expectancy (ESP)	3.13	2.89	2.81	3.03

**Note:**

Sample A: 2000–2004; Sample B: 2012–2014. Values are provided in years. Average lifespan is provided as mean ± standard deviation.

**Figure 5:**
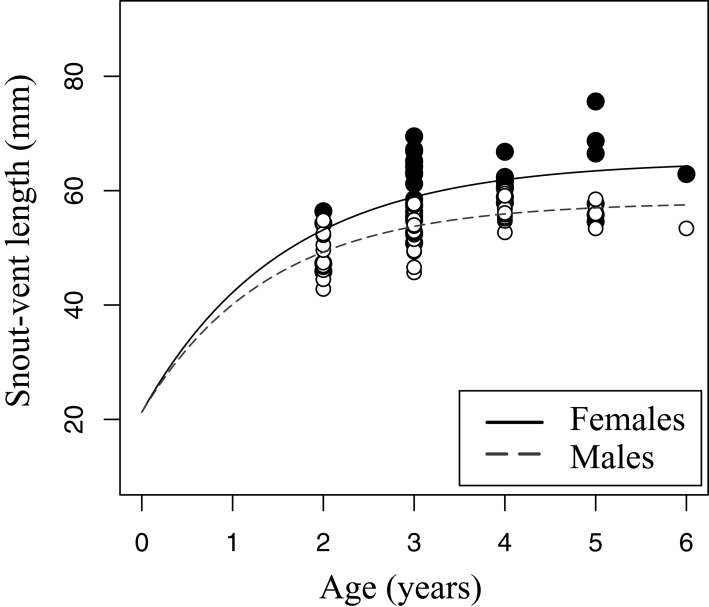
Growth pattern of *Pelobates fuscus* males and females within sample B (2012–2014). The growth pattern was computed following von Bertalanffy’s equation, using size-at-age data from sample B. Males (*n* = 52) are represented with white circles and dashed line, and females (*n* = 42) with black circles and continuous line.

The relation between age and SVL fitted von Bertalanffy’s growth model in both sexes (sample B, Fig. 5). The asymptotic average SVL was significantly higher in females (males: SVL_max_ = 58.0 mm, CI 95% [55.4–60.5]; females: SVL_max_ = 65.1 mm, CI 95% [59.6–70.7]), while the growth rate coefficient was similar in both sexes (males: *k* = 0.72, CI 95% [0.51–0.92]; females: *k* = 0.65, CI 95% [0.34–0.97]).

**Figure 6:**
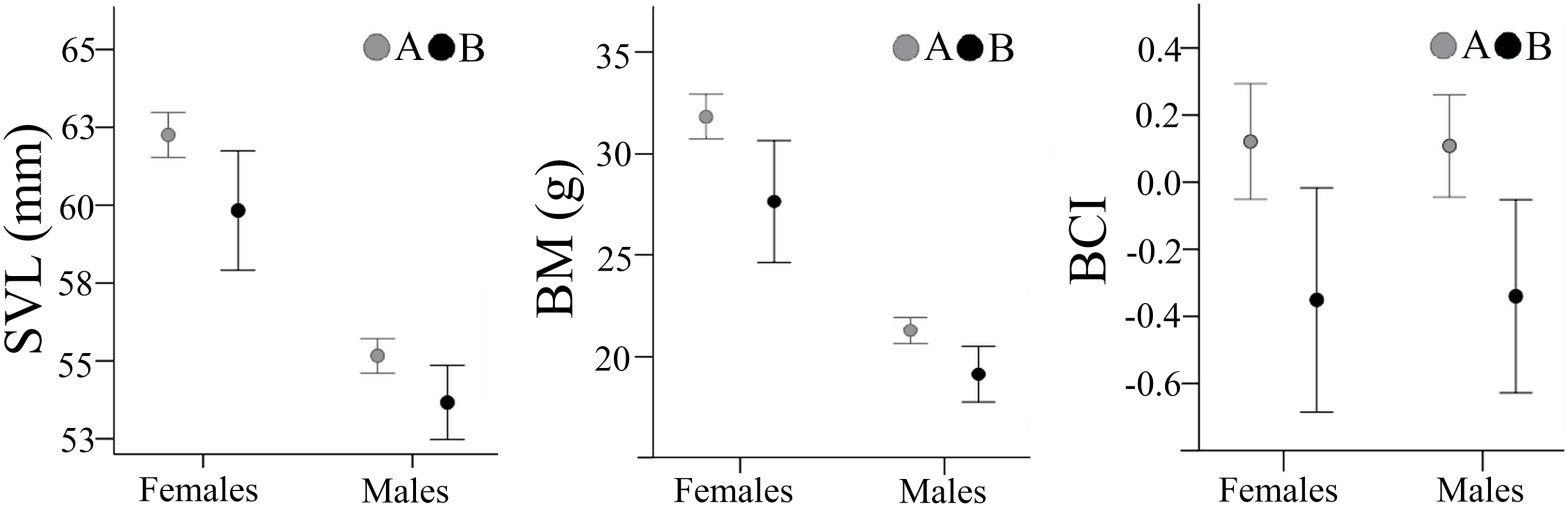
Body size and body condition of male and female *Pelobates fuscus*, at the two sampling moments—A(2000–2004) and B (2012–2014). SVL, snout-vent length (mm), BM, body mass (g), BCI, body condition index. Error bars represent 95% confidence intervals.

## Discussion

We found that, in the studied common spadefoot toad population, individuals responded to habitat degradation by a significant decrease in size and body condition, and a rather subtle decrease of average lifespan. The less obvious change expected in age-related parameters could be caused by the rather exponential loss of suitable habitat cover (pasture), and might become more evident over the span of several generations after the second sampling period. Thus, we suggest that changes in size parameters better reflect an immediate population response to habitat loss and fragmentation in common spadefoot toad populations and should be integrated into early-warning tools for detecting amphibian population declines, as previously highlighted by other similar research (e.g., Janin, Léna & Joly, 2011).

We estimate that the documented reduction in body condition will affect reproductive output and success, which could in turn result in decreasing population size. While a lower population density could diminish the competition for food resources and reduce the parasitic and pathogen load in natural, undisturbed ecosystems (e.g., Dietz, 1988), this might not be the case in ecosystems already disturbed by urbanization (Cordier et al., 2021). Moreover, local amphibian populations often face multiple threats that act in synergy and are being fueled by anthropogenic development, such as climate change and alien species. Thus, we expect severe consequences that would ultimately lead to the local extinction of the studied population, unless the remaining suitable natural habitat (pasture) is maintained. However, since the study area is not included in a designated natural protected site (e.g., Natura 2000), and given the continuous residential development, we also expect that the remaining habitats will be ultimately engulfed and transformed by the municipality.

Body size is a critical indicator of resource use and is tightly linked to individual fitness, offspring size, annual growth rate and lifespan (Angilletta, Steury & Sears, 2004). Although many species are resilient to some degree of change in their environment, the often rapid and extensive nature of anthropogenic changes can exceed their adaptive response abilities. For many animals, a change in behavior is very often the first response to human-altered conditions, since it can potentially improve an organism’s prospects of surviving and reproducing in a changing world (Wong & Candolin, 2015). Phenotypic plasticity, i.e., the ability of a particular genotype to express different phenotypes under altered environmental conditions (Thibert-Plante & Hendry, 2011), also acts as an adaptation in coping with changes. While both behavioral changes and phenotypic plasticity can buffer the impact of major environmental changes, these responses usually involve shifts in life-history traits. For example, in some commercially-exploited fish, intense size-selective harvest leads to slower growth rates, earlier maturation at smaller sizes, and increasing reproductive investment (Feiner et al., 2015). In turn, the decrease in size often increases mortality due to predation, causing a decline in biomasses and catches (Audzijonyte et al., 2013).

Life-history theory postulates links between age at sexual maturity, longevity, body size and reproductive investment (Stearns, 2000; Roff, 2002). There is a trade-off between allocating available energy to reproduction, somatic maintenance and growth (Charnov, Turner & Winemiller, 2001; Amat & Meiri, 2018). In some environments, individuals must mature earlier to increase their reproductive success, but they do so at the cost of a smaller body size. For example, in a study of the mountain frog *Nanorana quadranus* (Liu, Hu & Yang, 1960) inhabiting a wide range of habitats with different harshness levels, age structure did not differ between populations, but body size changed significantly (Wang et al., 2019). A study of the geographic variation in average age, body size and reproductive investment linked to variation in temperature and rainfall in the Australian frog *Crinia pseudinsignifera* Main, 1957 showed a variable response, where frogs from harsher environments invested less in their first reproductive event, grew older than their counterparts and achieved a larger body size (Reniers et al., 2015). In a population of Siberian toads *Strauchbufo raddei* (Strauch, 1876), exposed to heavy metal pollution, males increased reproduction investment (i.e., improved advertisement call and secondary sexual characteristics) at the cost of reduced health and longevity, a trade-off that might lead to a population decline (Guo et al., 2018). Jennette, Snodgrass & Forester (2019) found differences in size but not age, among the breeding populations of *Lithobates sylvaticus* (LeConte, 1825) and *Anaxyrus americanus* (Holbrook, 1836) along an urbanization gradient, while in contrast, Sinsch et al. (2007) found that age at maturity in males, and longevity and potential reproductive lifespan in females were significantly correlated to habitat quality in *Bufotes viridis* (Laurenti, 1768). These studies, as well as our results, suggest that age-related parameters could be rather discrete, species-specific indicators of life-history trade-offs caused by environmental changes of human origin. While our datasets did not allow for a comparison of growth patterns over time, we still provide baseline data regarding the growth parameters of males and females during the second sampling period, useful for further monitoring studies.

In animals with a complex life cycle like amphibians, changes in both the aquatic and terrestrial habitats have an influence on their body condition, growth and survival (Thompson & Popescu, 2021). The carryover effects of aquatic stressors are often difficult to disentangle from the impact of terrestrial habitat changes and the overall climate-change induced modifications. In addition, increased anthropic eutrophication of the breeding ponds might further promote amphibian diseases (e.g., Johnson et al., 2007). Since we did not assess reproductive success and size at metamorphosis (e.g., Székely et al., 2020), we could not separate the impact of changes in water and land quality and availability in the studied population, but we rather assessed an overall impact of landscape changes, which affected both the aquatic and nearby terrestrial habitats.

As urban environments become increasingly dominant in contemporary landscapes, they cause profound and complex environmental changes that induce fast and pronounced ecological and evolutionary changes in many species (Brans & De Meester, 2018). The response to urbanization include significant trait shifts: plant species show changes in flowering time, size and seed production (e.g., Palmat et al., 2016), birds shift egg laying periods and mating behaviour (e.g., Møller et al., 2015), water fleas (*Daphnia magna*) shift towards a faster pace of life (e.g., Brans & De Meester, 2018), while invertebrate habitat specialist species shift towards smaller body sizes (e.g., Magura, Ferrante & Lövei, 2020). These shifts also have an inevitable impact on the trophic resources of amphibians and recent studies have highlighted the rapid and alarming decline in insect biomass and diversity throughout Europe and worldwide during the last decades (e.g., Sánchez-Bayo & Wyckhuys, 2019; Wagner, 2020). Since insects are a major component of spadefoot toads diet (Cogălniceanu et al., 1998), we expect that a decrease in insect biomass and availability could affect their body condition and lower winter survival.

In this context, it is important to identify the most suitable monitoring tools that will detect early changes in population health before severe declines occur. Amphibian populations, due to their sensitivity to environmental stress, are often used in biomonitoring, and as such a variety of both invasive and non-invasive tools are available for estimating and measuring the impact of human activities, like fluctuating asymmetry (e.g., Niemeier, Mueller & Roedel, 2019), micronuclei tests (e.g., Bosch, Gorla & Aiassa, 2011), immunocompetence (e.g., Brown, Shilton & Shine, 2011), stress physiology (Narayan et al., 2019) or measures of heterozygosity (Eterovick et al., 2016). Our study showed that body measurements that require minimal handling of the animal and can be done on site provide a reliable measure of the effects of habitat loss and fragmentation. Moreover, age assessment showed a decrease in the average lifespan of the common spadefoot toad population in this study. Although changes in age parameters were less obvious in this case, it should be considered that these changes could not be evaluated separately in males and females between the two sampling moments (i.e., reduced and increased urban development), and previous studies showed that at least some age parameters can be sex-dependent in this species (Eggert & Guyétant, 1999; Cogălniceanu et al., 2014).

## Supplemental Information

10.7717/peerj.11678/supp-1 Supplemental Information 1 Raw dataset of size parameters in the studied *Pelobates fuscus* population, at two moments in time. Sample A = measurements collected during 2000–2004; Sample B = measurements collected during 2012–2014; SVL = measurements of snout-vent length, in mm; BM = measurements of body mass, in g.

10.7717/peerj.11678/supp-2 Supplemental Information 2 Raw dataset of age in the studied *Pelobates fuscus* population, at two moments in time. Sample A = toes collected during 2000–2004; Sample B = toes collected during 2012–2014; Age = number of lines of arrested growth (LAGs) corresponding to 1 year of life.

## Additional Information and Declarations

### Field Study Permissions

The following information was supplied relating to field study approvals (i.e., approving body and any reference numbers): Field sampling received approval through Ministery Order No. 1173.2010 (in OM, part I, No. 609/27.VIII.2010).

### Data Availability

The following information was supplied regarding data availability: The raw measurements for body size (snout-vent length and body mass) and age assessments are available in the Supplemental Files.
